# Systematic Evaluation of Image Tiling Adverse Effects on Deep Learning Semantic Segmentation

**DOI:** 10.3389/fnins.2020.00065

**Published:** 2020-02-07

**Authors:** G. Anthony Reina, Ravi Panchumarthy, Siddhesh Pravin Thakur, Alexei Bastidas, Spyridon Bakas

**Affiliations:** ^1^Intel Corporation, Santa Clara, CA, United States; ^2^Center for Biomedical Image Computing and Analytics, University of Pennsylvania, Philadelphia, PA, United States; ^3^Department of Radiology, Perelman School of Medicine, University of Pennsylvania, Philadelphia, PA, United States; ^4^Department of Pathology and Laboratory Medicine, Perelman School of Medicine, University of Pennsylvania, Philadelphia, PA, United States

**Keywords:** segmentation, tiling, deep learning, CNN, brain tumor, glioma, BraTS, satellite imaging

## Abstract

Convolutional neural network (CNN) models obtain state of the art performance on image classification, localization, and segmentation tasks. Limitations in computer hardware, most notably memory size in deep learning accelerator cards, prevent relatively large images, such as those from medical and satellite imaging, from being processed as a whole in their original resolution. A fully convolutional topology, such as U-Net, is typically trained on down-sampled images and inferred on images of their original size and resolution, by simply dividing the larger image into smaller (typically overlapping) tiles, making predictions on these tiles, and stitching them back together as the prediction for the whole image. In this study, we show that this tiling technique combined with translationally-invariant nature of CNNs causes small, but relevant differences during inference that can be detrimental in the performance of the model. Here we quantify these variations in both medical (i.e., BraTS) and non-medical (i.e., satellite) images and show that training a 2D U-Net model on the whole image substantially improves the overall model performance. Finally, we compare 2D and 3D semantic segmentation models to show that providing CNN models with a wider context of the image in all three dimensions leads to more accurate and consistent predictions. Our results suggest that tiling the input to CNN models—while perhaps necessary to overcome the memory limitations in computer hardware—may lead to undesirable and unpredictable errors in the model's output that can only be adequately mitigated by increasing the input of the model to the largest possible tile size.

## 1. Introduction

Since their resurgence in 2012 convolutional neural networks (CNN) have rapidly proved to be the state-of-the-art method for computer-aided diagnosis in medical imaging, and have led to improved accuracy in classification, localization, and segmentation tasks (Krizhevsky et al., 2012; Chen et al., 2016; Greenspan et al., 2016). However, memory constraints in deep learning accelerator cards have often limited training on large 2D and 3D images due to the size of the activation maps held for the backward pass during gradient descent (Chen et al., 2016; Ito et al., 2019). Two methods are commonly used to manage these memory limitations: (i) images are often down-sampled to a lower resolution, and/or (ii) images are broken into smaller tiles (Huang et al., 2018; Pinckaers and Litjens, 2018). Tiling is often applied when using large images due to the memory limitations of the hardware (Roth et al., 2018). Specifically, in CNN models, the activation maps of the intermediate layers use several times the memory footprint of the original input image. These activation maps can easily increase the allocated memory to hundreds of gigabytes. Fully convolutional networks are a natural fit for tiling methods, as they can be trained on images of one size and perform inference on images of a larger size by breaking the large image into smaller, overlapping tiles (Ronneberger et al., 2015; Çiçek et al., 2016; Roth et al., 2018). To perform the overlapping tiling at inference time, varying *N* × *N* (or in the 3D case, *N* × *N* × *N*) tiles are cropped from the whole image at uniformly spaced offsets along the image dimensions.

Tiling introduces additional model hyperparameters—namely, tile size, overlap amount, and aggregation process (e.g., tile averaging/rounding)—that must be tuned to generate better predictions. For example, Roth et al. performed abdominal organ segmentation on 512 × 512 CT images with between 460 and 1,177 slices by using input tiles of size 132 × 132 × 116 to yield output prediction tiles of 44 × 44 × 28 in a Cascaded 3D U-Net (Roth et al., 2018). In the second stage of the prediction, the probabilities for overlapping tile predictions were averaged to produce a better *Dice* Coefficient result. Zeng and Zheng (2018) introduced “Holistic Decomposition Convolution” that—when added to a conventional 3D U-Net—significantly reduced the size of the input data while maintaining the useful information for the semantic segmentation. They compared the effects of 50 × 50 × 40, 96 × 96 × 96, and 200 × 200 × 40 tile crops from a 480 × 480 × 160 MR and determined that they had better *Dice* Coefficient, Hausdorff Distance, and Average Surface Distance when using the largest tile size that could fit into memory. Isensee et al. (2019) used a sliding window with a half-tile overlap and test-time data augmentation that mirrored the tile along all axes. They also favored larger tile size over large batch size in order to “maximize the amount of spatial context that can be captured.” Ghosh et al. (2018) found that by rotating or flipping the input tile, the prediction was slightly different for the same tile. By averaging these small variations in the tiled predictions, Ghosh produced improved predictions in structures within satellite imagery from a dilated U-Net topology. Huang et al. determined that zero-padding and strided convolutions (i.e., stride > 1)—two methods commonly used in CNNs—created variability in predictions close to the tile border and caused translation variance in the output prediction (Huang et al., 2018).

Previous works like these refer to tiling methods as “necessary due to constraints in memory” rather than methods to “improve the accuracy of the algorithms” (Chen et al., 2016; Roth et al., 2018; Isensee et al., 2019; Ito et al., 2019). In other words, the tiling method compensates for insufficient memory rather than adds predictive power. If more memory were available for training and inference of these models, then tiling methods would have not been necessary or even desirable. For example, Kamnitsas et al. (2017) created the first state of the art 3D topology for predicting brain tumors by finding tiles of “image-segments” which are “larger than individual patches [tiles], but small enough to fit into memory.” Roth et al. (2018) remarked, “with the growing amount of …memory, overlapping sub-volume predictions …will be reduced as it will be come possible to reshape the network to accept arbitrary 3D input image sizes.”

In this study, we focus on the tiling approach—during both model training and model inference—and its influence on the model prediction. We implemented U-Net topologies for both 2D (Ronneberger et al., 2015) and 3D (Çiçek et al., 2016) data, and we question whether this image tiling approach is indeed as accurate as simply performing inference on the whole image. In a previous report (Reina and Panchumarthy, 2018), we noticed that using the entire 2D image gave better predictions than the tiling approach for a 2D U-Net model trained to detect glial tumors from brain magnetic resonance imaging (MRI). In this study, we extend those results by systematically (i) evaluating the resulting effects in both medical and non-medical data, (ii) comparing both 2D and 3D U-Net models, and (iii) suggesting that these differences are caused by operations within the CNN model that vary due to translations in the input of the model. Finally, we show that these issues can be partially addressed by increasing the size of the tile—up to and including training and inferring on the whole image.

## 2. Methods

### 2.1. Data

#### 2.1.1. Brain Tumor Segmentation (BraTS)

**Figure 1:**
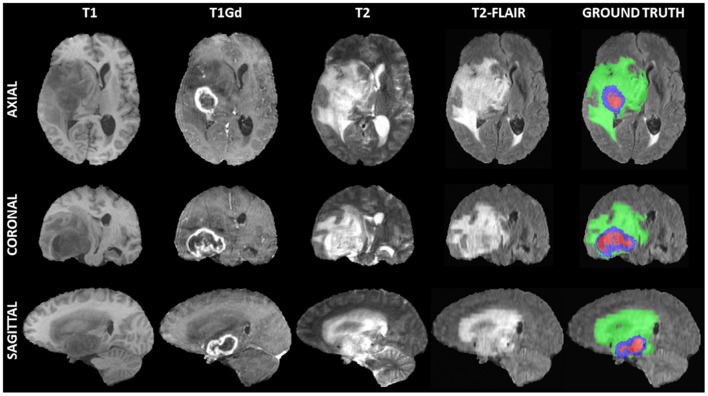
Example of a 3D input multi-parametric Magnetic Resonance Imaging scan from the International Brain Tumor Segmentation (BraTS) challenge. From left to right all four input modalities are illustrated, including native T1-weighted (T1), T1 post-contrast (T1Gd), native T2-weighted (T2), and T2 Fluid Attenuated Inversion Recovery (T2-FLAIR), followed by the ground truth expert annotation of all three tumor sub-regions, provided as part of the BraTS dataset. From top to bottom three views (i.e., Axial, Coronal, Sagittal) of these 3D volumes are depicted to showcase the 3-dimensional nature of these scans.

The medical data used for our evaluations reflect the publicly-available training dataset of the International Brain Tumor Segmentation (BraTS) challenge 2019^1^ (Figure 1) (Menze et al., 2014; Bakas et al., 2017a,b,c; Bakas et al., 2018). BraTS created a publicly-available multi-institutional dataset for benchmarking and quantitatively evaluating the performance of computer-aided segmentation algorithms for brain tumors from MRI scans. These scans were acquired by 1T, 1.5T, or 3T MRI scanners and all the ground truth labels were manually approved by expert, board-certified neuroradiologists. The dataset we used here comprises pre-operative multi-parametric MRI scans from 335 patients diagnosed with glioma. The exact modalities of the mpMRI scans included describe native T1-weighted (T1), post-contrast T1-weighted (T1Gd), T2-weighted, and T2 Fluid Attenuated Inversion Recovery (FLAIR) scans. We randomly split this dataset into 270 training, 30 validation, and 35 testing scans.

Although the BraTS data describe 3D MRI scans, here we are considering the 155 2D slices from each scan to be an independent image for training a 2D model. However, all 2D slices from a single patient scan were contained in only one of the three dataset splits (training/validation/testing), to prevent any potential data leakage toward learning data co-linearities. Specifically, there were 41,850, 4,650, and 5,425 2D image/mask pairs corresponding to 270, 30, and 35 3D MRI scans, across the training, validation, and testing sets, respectively. All 2D images were Z-scored along the channel axis from pre-computed means and standard deviations of the 3D MRI scan. The original 2D slices were 240 × 240 pixels (i.e., whole image).

#### 2.1.2. SpaceNet Vegas Satellite Imagery

**Figure 2:**
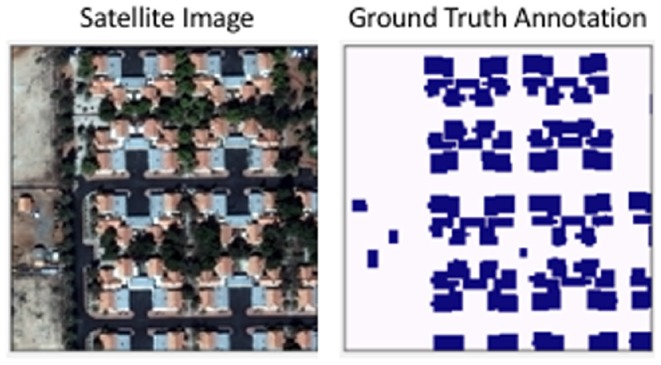
An example of the SpaceNet-Vegas images used in this study. The ground truth annotations for buildings and other structures were professionally labeled.

The non-medical data is sourced from the public SpaceNet satellite imagery dataset suite (Figure 2) (SPA, 2018; Weir et al., 2019)^2^. Specifically, we used the Vegas subset of the data (SN-Vegas). It is comprised of 3,851 30 cm spatial resolution, pan-sharpened, RGB satellite imagery over the city of Las Vegas, Nevada (USA) as well as latitude-longitude annotations for 108,942 building footprint polygons within the city. We exclude the official competition test dataset from this study because it does not contain publicly-available ground truth annotations. The images were captured by WorldView-2 and 3 satellites, and filtered to exclude images with excessive cloud cover as well as extreme capture angles. The labels were professionally created by geospatial data labeling vendor Radiant Solutions^3^.

The SpaceNet-Vegas dataset was split into 70% training (2,695 images), 20% validation (770 images), and 10% testing (386 images), corresponding to 77,099 training, 21,505 validation, and 10,338 testing building polygons. All inputs were Z-scored along the channel axis from pre-computed means and standard deviations. All training inputs were also subject to random horizontal and vertical flips, and rotations between 0 and 360°.

### 2.2. U-Net Topology

**Figure 3:**
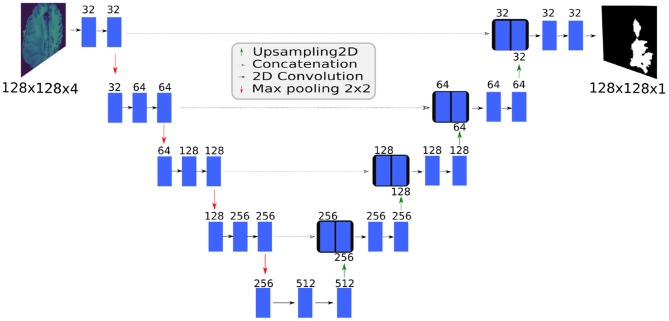
The BraTS 2D U-Net topology. The SpaceNet 2D U-Net and the BraTS 3D U-Net topologies have similar architectures.

U-Net is a fully convolutional network based on an encoder-decoder architecture (Figure 3). The contracting path captures context and the expanding path enables localization. Unlike the standard encoder-decoder, each feature map in the expanding path is concatenated with a corresponding feature map from the contracting path, augmenting downstream feature maps with spatial information acquired using smaller receptive fields. Intuitively, this allows the network to consider features at various spatial scales. By design, U-Net is agnostic to image size, and its training and inference can be performed on images of different size.

### 2.3. 2D U-Net for Medical Data (BraTS)

We adapted a 2D U-Net model for training on the BraTS data, and specifically used four MRI modalities as input and output an equivalently-sized mask predicting the whole tumor appearing in a 2D slice.

#### 2.3.1. Architectural Modifications

In favor of allowing wider reproducibility of our results, we specifically modified the originally published 2D U-Net topology by reducing the number of feature maps by half (from 64 in the first convolutional layer down to 32) and adding dropout (0.2) just before the 3rd and 4th max pooling layers. We also used zero padding in all convolutional layers to maintain the image dimensions and eliminate the need to crop the image for concatenation. The reduction of the originally proposed feature maps happened in favor of our results been reproducible by others without requiring extreme hardware equipment.

#### 2.3.2. Training Process

We implemented the model used here in Keras 2.2.4 and TensorFlow 1.11, and made the complete source code publicly available^4^. Stochastic gradient descent with the Adam optimizer (learning rate = 1e-4) was used to minimize the loss function −log(*Dice*), where *Dice* is defined as in equation 1 on page 6. A batch size of 128 was used during training. We created a batch generator which randomly selected cropped images/masks from the training set for each batch.

The 2D model was trained for 40 epochs. During training, a random crop of 128 × 128 pixels was taken from the normalized 2D images and their corresponding ground truth masks. Randomized flipping (up/down and left/right), and 90 degree rotation of the training set images were also used during online data augmentation. The *Dice* on a center 128 × 128 crop of the validation dataset was calculated after every epoch. The model that produced the highest *Dice* on the center 128 × 128 crop of the validation data was considered the best trained model.

For pre-processing of the images, on a per image basis, images were clipped to 98 percentile of their values and standardization was applied only on non-zero pixels making background consistent over all images. This created a consistent effect of normalization over the images.

#### 2.3.3. Zero Padding Experiments

We conducted additional experiments to determine the effects of zero padding on the tiling approach. This stemmed from the findings of Huang et al. (2018), who suggested that zero padding used in CNN topologies caused variability in predictions at the tile border. To assess this, we also created and trained an additional 2D U-Net model that did not include zero-padding for any of the convolutional layers. We named this the “no pad BraTS” model.

The “no pad BraTS” model was trained in the same way as the first 2D U-Net model, but with the following changes. This “no pad BraTS” model took as input a random crop of 236 × 236 and output a 52 × 52 prediction. The decrease in the output size was due to the progressive loss in the border pixels after each non-padded convolutional layer (Ronneberger et al., 2015). The input size was chosen to be slightly smaller than the whole 240 × 240 slice so that we could evaluate if the prediction changed with small translations of the input. The model prediction was compared to a similarly-cropped version of the ground truth mask. It was trained for 40 epochs and the model that produced the highest *Dice* on the validation data was considered the best trained model.

#### 2.3.4. Inferring on 2D Tiles (Tiling Approach)

**Figure 4:**
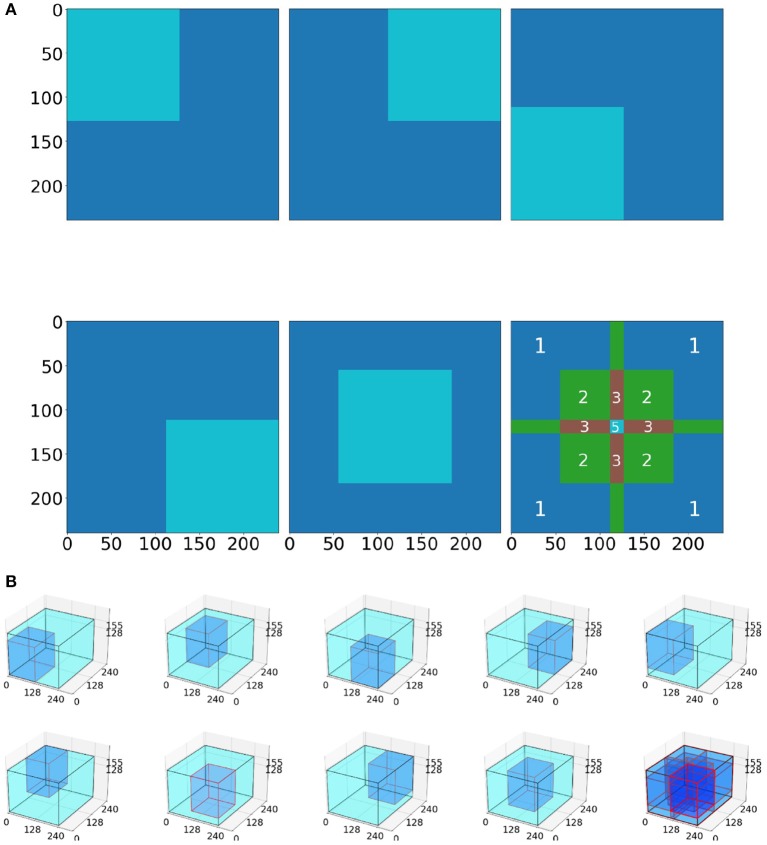
Tiling process schematic. **(A)** In the 2D model, five tiles (4 corners, 1 center) are averaged to produce the whole image prediction. The 3rd picture in the 2nd row depicts the intensity of the tile overlapping. Notably, the tile predictions are either (i) first rounded and then averaged together, or (ii) first averaged together and then rounded. **(B)** Example for the 3D BraTS model, where the tiling algorithm is similar with the 2D, but this time uses nine tiles (8 corners and 1 center).

Inference was performed individually on five 128 × 128 pixel-sized tiles, extracted from the four corners and the center of the slice (Figure 4A). We performed inference on the whole 2D slice using the model and then stacked the 155 slices on a per scan basis to generate a predicted 3D segmentation mask of the entire scan.

We utilized and compared two tiling aggregation approaches. The first approach, *rounding after averaging*, is described by Roth et al. (2018). In our case the predictions from these five 128 × 128 tiles were first averaged and then rounded to either 0 or 1 (threshold:0.5). We compared the *rounding after averaging* approach with a *rounding before averaging* approach: The five 128 × 128 tiles were rounded to either 0 or 1 (threshold:0.5) and then averaged to provide the whole image prediction (*rounding before averaging*). Slicewise predictions for each patient scan were then stacked together to compare the 2D predictions with predictions from the 3D BraTS model.

#### 2.3.5. Inferring on the Whole 2D Slice

For fully-convolutional topologies, the TensorFlow model can be created with a run-time defined height and width by specifying the input dimensions to be [*Height, Width, Channels*] = [*None, None*, 4] where *None* describes the run-time defined parameter^5^.

By defining and training the model in this manner, we can pass an image of almost any size into the model and perform inference. The only limitation to the input image size is that the dimension must be divisible by 2^4^ in order to align with the 4 max-pool layers of the U-Net model and correctly concatenate the skip connections.

### 2.4. 3D U-Net for Medical Data (BraTS)

To create the 3D U-Net model, we used the same number of convolutional and max-pooling layers as we used in the 2D U-Net model (Figure 3). We altered the implementation of the originally proposed 3D U-Net model (Çiçek et al., 2016) by replacing the ReLU layer with a leaky ReLU activation and adding instance normalization after each leaky ReLU (Xu et al., 2015; Ulyanov et al., 2016).

We further modified this implementation by using an initial learning rate of 0.01. A learning rate decay factor of 0.5 was applied when the value of the validation loss had not been in the five best previous losses (i.e., check_best = 5). Training stopped when the validation loss did not improve in the past 20 epochs (i.e., patience = 20). Finally, the weights that yielded the lowest validation loss were used for the final model.

The 3D BraTS model is trained for 100 epochs on 9 tiles, of 128 × 128 × 128 voxels, cropped from the 8 corners and the center of a 3D MRI scan (Figure 4B).

Inferring via a tiling approach was also performed similar to the 2D U-Net case (section 2.3.4), but used 128 × 128 × 128 tiles from the eight corners and the center of the whole image (Figure 4B). These nine 128 × 128 × 128 tiles were averaged to provide a prediction of the whole mask.

Whole image inference was also performed similar to the 2D U-Net (section 2.3.5) but using the whole 240 × 240 × 155 scan.

### 2.5. 2D U-Net on Satellite Data (SpaceNet-Vegas)

The SpaceNet model uses a single satellite image from SpaceNet-Vegas as input, and outputs an equivalently-sized mask predicting the building footprints.

#### 2.5.1. Architectural Modifications

The originally published topology was modified by introducing batch normalization to the output of a convolution layer, prior to the activation, for regularization purposes.

#### 2.5.2. Training Process

All models were trained for 300 epochs using the Adam optimizer with a 5e-4 learning rate to optimize the Binary Cross Entropy loss. To test our hypothesis that a model trained in the whole image outperforms a tiling-based approach, we followed two training processes here; based on (a) tiling, and (b) down-sampling.

For models trained via tiling, the input image's source resolution of 650 × 650 is maintained and a random crop of the desired dimension is selected. Different models were trained for each of the following random tiling sizes:

- 128 × 128
- 256 × 256
- 384 × 384
- 496 × 496

Due to the U-Net architecture, the input dimensions to the model must be divisible by 2^5^ in order to align with the 5 max-pool layers. Consequently, for the models trained on the entire image via down-sampling, the original image was downsampled with anti-aliasing and bilinear interpolation to 512 × 512 and 640 × 640.

#### 2.5.3. Zero Padding Experiments

As with the BraTS experiments, we created additional SpaceNet experiments to determine the effects that zero padding had on the tiling approach. We also created and trained additional SpaceNet models that did not include zero-padding for any of the convolutional layers (namely the “no pad SpaceNet” models).

#### 2.5.4. Inferring on 2D Tiles

Inference was performed using tiles of the same size that was used when training the model, with a 50% overlap between tiles in both the vertical and horizontal dimension. The overlapping tiles were averaged to provide the whole image prediction.

### 2.6. Evaluation Metric

#### 2.6.1. …for the Medical Data

In consistency with the metric used in the BraTS challenge, the Dice Similarity Coefficient (*Dice*) was used here to measure the quality of the tumor predictions. *Dice* is defined as:

(1) $$\begin{array}{l} Dice=\frac{2\times TP}{2\times TP+FP+FN} \end{array}$$

where *TP, FP, TN, FN* are the number of True Positive, False Positive, True Negative, and False Negative pixels.

#### 2.6.2. …for the Satellite Data

**Figure 5:**
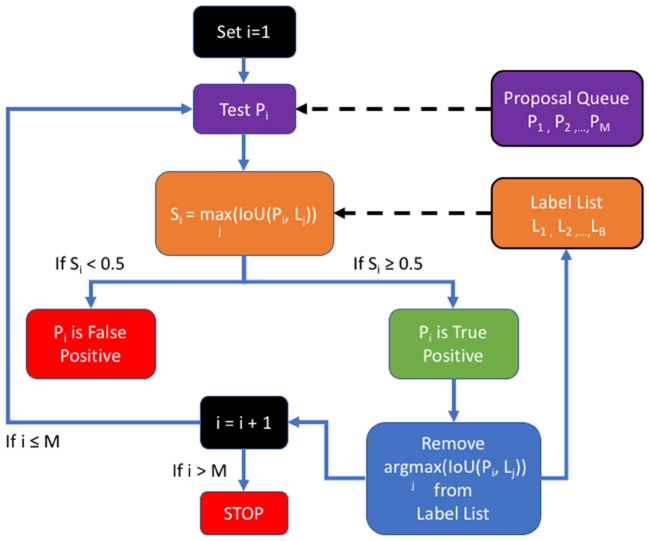
The SpaceNet *F*1 metric: a list of proposals is generated by the detection algorithm and compared to the ground truth in the list of labels.

In order to measure performance relative to established benchmarks on SpaceNet, we used the post-processing Polygon *F*1 metric, displayed in Figure 5; namely, the predicted segmentation mask is polygonized based on same-value pixel connectivity to generate a set of proposed polygons in latitude and longitude space. We then calculate the spatial intersection over union (i.e., Jaccard Index) between proposed and ground truth polygons. A true positive is asserted if the Jaccard value is above 0.5. Once we establish TP, FP, and FN counts, we compute the *Dice* (also known as SpaceNet (polygonal) *F*1 Score)—the harmonic mean between precision and recall—over these matched polygons and compare this metric to the *Dice* calculated on a pixelwise basis (Hagerty, 2016).

## 3. Results

### 3.1. 2D BraTS Model

The best trained 2D BraTS model yielded an average *Dice* of 0.8877 when inferred on a single center 128 × 128 tile of the test dataset slices. Furthermore, as explained in the methods, although this model was trained on random 128 × 128 tiles, we were able to perform inference on the entire 240 × 240 2D image slice. The whole 2D slice predictions resulted in an average *Dice* on 0.8743 on the whole 3D volume. Using the 2D BraTS model with five 128 × 128 tiles, resulted in an average *Dice* of 0.8599 for the tiling aggregation method of *rounding after averaging*. Using the *rounding before averaging* tiling aggregation method, resulted in a 0.8998 average *Dice* (Table 1).

**Table 1:** Results of 2D U-Net on medical data (BraTS).

**Inference on:**	**Whole 2D slice**	**2D tiles**	**2D tiles**
Aggregation approach	N/A	(Rounding after averaging)	(Rounding before averaging)
*Dice*	0.8743	0.8599	0.8998

*Comparing whole 2D slice prediction to two tiling aggregation methods*.

**Figure 6:**
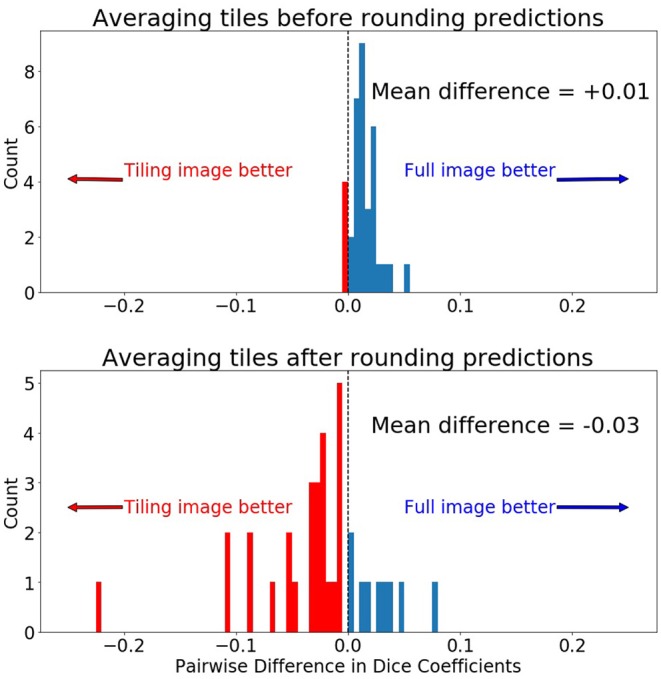
Comparing pairwise *Dice* differences between the prediction on the whole 240 × 240 image and the 128 × 128 tiles. Zero indicates both methods produced equal *Dice* scores for the same scan. **(Top)** Prediction was made by first averaging the 5 tiles and then rounding the final prediction to 0 or 1. **(Bottom)** Prediction was made by first rounding the tiled predictions to 0 or 1 and then averaging the predictions.

Collectively in the testing dataset, application of different tiling aggregation approaches (i.e., *rounding after averaging*, and *rounding before averaging*) revealed that when we aggregated the predicted segmentations by *rounding after averaging*, the 2D segmentations of individual subjects were inferior to the segmentations obtained from the 3D model. Contrarily, evaluation of the tiling aggregation approach, where *rounding before averaging* was applied, yield that on average more 2D predictions were closer to the ground truth than when using the 3D model (Figure 6).

### 3.2. 3D BraTS Model

The results of the 3D U-Net BraTS model showed a different behavior when compared with the results of the 2D U-Net model. Specifically, there were no significant differences observed when the predictions of the model inferred on the whole 3D MRI scan were compared to the predictions of any of the tiling aggregation approaches. Inferring the 3D BraTS model on the whole 3D scan resulted in an average *Dice* of 0.8974, when for the tiling aggregation method of *rounding after averaging* and of *rounding before averaging* the average *Dice* was equal to 0.8991 and 0.8984, respectively (Table 2).

**Table 2:** Results of 3D U-Net on medical data (BraTS).

**Inference on:**	**Whole 3D scan**	**3D tiles**	**3D tiles**
Aggregation approach	N/A	(Rounding after averaging)	(Rounding before averaging)
Average *Dice* (±σ)	0.8974 (± 0.0702)	0.8991 (± 0.0666)	0.8984 (± 0.0670)

*Comparing whole 3D scan prediction to two tiling aggregation methods*.

### 3.3. 2D SpaceNet-Vegas

With the satellite image dataset, we note that higher accuracy was obtained by training on a larger tile size (i.e., larger context of the image). The model trained on 128 × 128 random tiles and inferred on the whole 650 × 650 image with 128 × 128 sliding tiles, resulted in a *Dice* score of 0.791, whereas the model trained on the whole 2D image resized to 640 × 640 and inferred on the whole 650 × 650 image resulted in a *Dice* score of 0.917. To train on the whole image, we interpolated the image to 640 × 640 as the U-Net topology require the input image to be multiple of 2^5^ to align with 5 max-pool layers. Tables 3, 4 denote that both the evaluation metrics of *Dice* and SpaceNet *F*1 (polygon-wise and computed over the entire dataset, not per image) improve as the training tile size increases.

**Table 3:** Results of 2D U-Net with zero-padding on non-medical data (SpaceNet Vegas).

**Tile size**	**128 × 128**	**256 × 256**	**384 × 384**	**496 × 496**	**512 × 512**	**640 × 640**
	**crop**	**crop**	**crop**	**crop**	**interp**	**interp**
*Dice*	0.873	0.900	0.896	0.918	0.917	0.918
SpaceNet *F*1	0.748	0.803	0.800	0.838	0.840	0.847

*Dice and SpaceNet (polygon-wise Dice) F1 metrics on varying tile size*.

**Table 4:** Results of 2D U-Net without zero-padding on non-medical data (SpaceNet Vegas).

**Tile size**	**128 × 128**	**256 × 256**	**384 × 384**	**496 × 496**	**512 × 512**	**640 × 640**
	**crop**	**crop**	**crop**	**crop**	**interp**	**interp**
*Dice*	0.865	0.896	0.907	0.918	0.912	0.914
SpaceNet *F*1	0.734	0.781	0.797	0.806	0.808	0.821

*Dice and SpaceNet (polygon-wise Dice) F1 metrics on varying tile size*.

## 4. Discussion

Our results denote substantial differences in our 2D U-Net architecture, both for medical and non-medical (i.e., satellite) data. Specifically, the evaluation of *Dice* show superiority when inferring our model in the whole 2D image, when compared with inferring in smaller image tiles, supporting our hypothesis for the large tile sizes. Furthermore, gradual increments of the tile sizes shows gradual improvement in the performance. Following the evaluation of our 3D U-Net model, we note that the performance on 3-dimensional data did not show substantial difference when comparing inference on the whole 3D image and inference on 3D tiles. We hypothesize that this happens due to the inclusion of large image context (e.g., more neighboring voxels) along the third dimension.

The overlapping tiling approach is commonly used by researchers to apply fully convolutional models on large 2D and 3D images that would ordinarily not fit into available memory (Chen et al., 2016; Roth et al., 2018). Isensee et al. (2019), for example, specifically designed their topology to “automatically set the batch size, tile size and number of pooling operations for each axis while keeping the memory consumption within a certain budget.” We suggest that researchers should be designing their topologies not to fit into a hardware constraint, but instead to produce the most accurate model possible.

**Figure 7:**
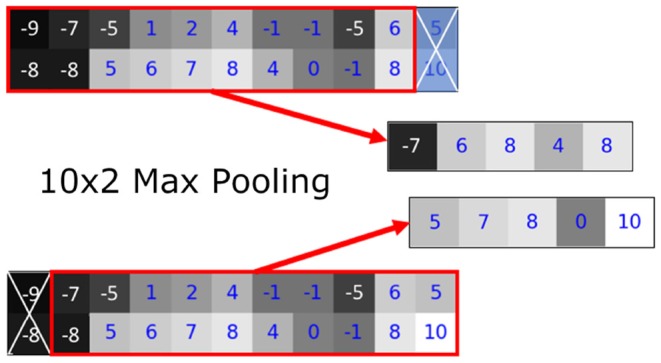
The translationally-variant nature of the MaxPooling layer: note how the result of MaxPooling significantly differs with a translation of one pixel to the 10 × 2 window between the top and bottom inputs even though they contain the same values (middle rows). Successive MaxPooling layers (or any non-unary, strided convolution) compound the effect because they effectively increase the receptive field window size. Hence, a model with three 2 × 2 MaxPooling layers would show translational variance for offsets of up to 2^3^ = 8 pixels.

**Figure 8:**
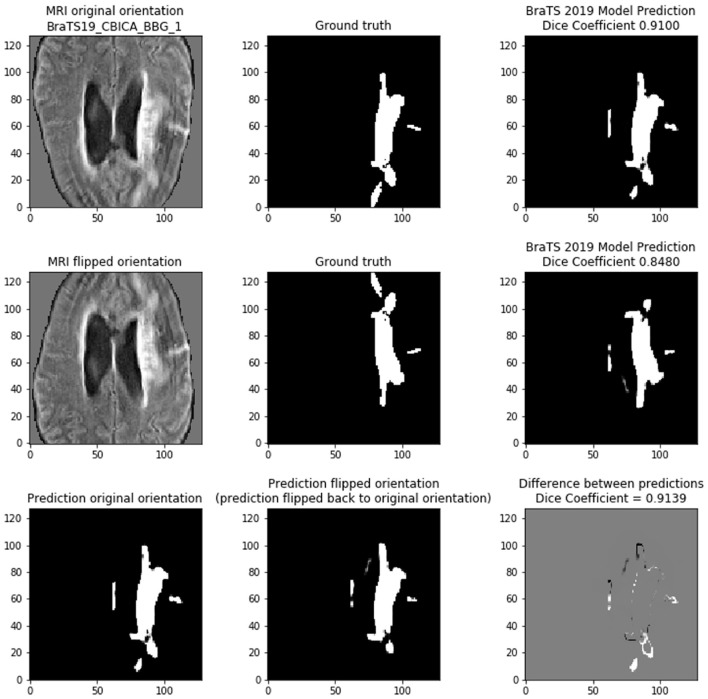
Demonstrating the variability of the 2D BraTS model. **(Top)** Prediction based on normal orientation of the MRI input. **(Middle)** Prediction based on vertical flip of the MRI input. **(Bottom)** Comparing the predictions of the normal and flipped inputs. The prediction of the flipped input was re-flipped to allow direct comparison with the normal orientation prediction. In bottom right figure, gray pixels indicate no difference, black pixels are in the flipped prediction but are not present in the normal prediction, and white pixels are in the normal prediction but are not present in the flipped prediction.

**Figure 9:**
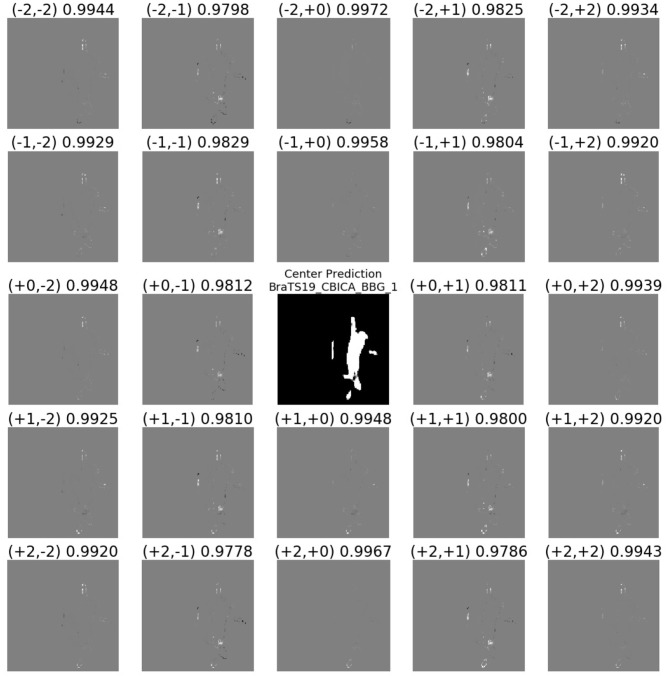
Demonstrating the translational variance of the 2D BraTS model. The center shows the prediction of the model on a 128 × 128 center crop of the MRI. Note the entire tumor fits within this tile. The surrounding subplots show the difference in the prediction as the MRI input crop is offset by one or two pixels in either dimension of the slice. The differences between the predictions in the overlapping pixels show that even the smallest translation in the input can create a difference in the output. Gray pixels indicate no difference in the predictions of the overlapping pixels between the center crop and the offset crop, black pixels are in the offset-crop prediction but are not present in the center-crop prediction, and white pixels are in the center-crop prediction but are not present in the offset-crop prediction.

We found that the variance in the prediction can be seen in the linear transformation (flipping) and affine transformation (translation) (**Figures 8**, **9**). Most neural networks include some component that makes it translationally-variant, such as a pooling layer or non-unary convolutional stride. In other words, the whole image is not necessarily the sum of individual tiles. In Figure 7, we demonstrate this effect due to a 2 × 2 max-pooling layer. Both the top and bottom use identical 11 × 2 arrays. If a 10 × 2 tile is used to perform the max-pooling, there are only two possible tiles. Notice that each tile produces different results. We further found that this behavior caused by the pooling layers most prominently affects the sharp intensity changes in object boundaries. We believe that many of our results on “blobbier” borders that are more sensitive to even minor affine transforms to the tiles are a result of these translationally-variant operations, especially the max-pooling operation.

Although these differences in prediction are often localized to the segmentation border, the boundaries of the tumor or buildings are often the most relevant to the task. Especially in medical imaging, ensuring adequate tumor margins are critical to successful therapeutic planning and treatment.

### 4.1. Medical Data (BraTS)

If the models were linear, then any linear transformation to the model input should result in the same prediction (with the same linear transformation). Figure 8 shows that on scan *BRATS19_CBICA_BBG_1.nii.gz* it achieves a *Dice* of 0.9100 on the center 128 × 128 tile of slice 94. However, if the MRI input is simply flipped vertically, then the prediction is changed. In this case, the *Dice* shows that the model provides a worse prediction with the flipped input (*Dice* = 0.8480). By reversing the linear transform (i.e., unflip the prediction) the two model predictions can be compared directly to show that they are indeed different (cross-prediction *Dice* = 0.9139). Although the two predictions are very similar, the bottom row of Figure 8 highlights the differences occur along the tumor borders. We find that the tumor borders appear to be where the predictions differ.

Figure 9 shows the translational variance of the model. The center shows the prediction of the model on a 128 × 128 center crop of the MRI. As the grid in the figure indicates, each tile shows the difference between pixels that overlap between the predictions of the center crop and a crop translated ±1 or 2 pixels in each dimension from the center crop. The *Dice* confirm that the overlapping predictions, while similar, differ significantly along the border of the tumor. This pattern of differences along the segmentation border was typical in the results. Note that the translations of (+2, +2) and (−2, −2) are a multiple of the max pooling stride and should be less sensitive to the translation (cf. Huang et al., 2018).

**Figure 10:**
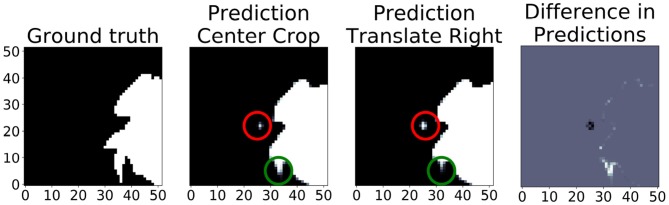
In this “no pad BraTS” model, there is still translational variance despite the model containing no zero padding in the convolutional layers. The “Prediction Center Crop” refers to the prediction when using a 236 × 236 center crop of the input slice. “Prediction Translate Right” refers to a similar crop but translated one pixel to the right of the center. The green and red circles highlight predictions that have changed due translating by a single pixel. The figure on the right shows the difference in the overlapping regions between the “Center Crop” and “Translate Right” predictions. Gray indicates no difference. White indicates a prediction in the “Center Crop” that was not in the “Translate Right.” Black indicates a prediction in the “Translate Right” that was not in the “Center Crop”.

Figure 10 shows the translational variance of the “no pad BraTS” model. In this case, the model was trained without a zero pad in the convolutional layers so that we could assess the effects of zero padding on the prediction output. In the figure, the “Center Crop” refers to a 236 × 236 center crop of the 240 × 240 slice and “Prediction Translate Right” refers to a crop that has been translated one pixel to the right of the center crop. When we compare the prediction regions that overlap, we find several areas where the tumor prediction has changed (red and green circles). This demonstrates that the translational invariance due to tiling cannot be mitigated by simply modifying the topology to only use valid pixels in the convolutional layers.

Application of different tiling aggregation approaches (i.e., *rounding after averaging*, and *rounding before averaging*) revealed unpredictable and inconsistent results. This introduces a new parameter to standardize the results. The user must be aware of this discrepancy and make appropriate conclusion by experimenting with different tiling aggregation methods. Furthermore, the results of the 3D U-Net model inference demonstrate that greater image context (3D vs. 2D) contributes in the performance, but also that after the inclusion of sufficient image context (i.e., when providing enough context) the model converges and no further improvements are observed.

The two different tiling aggregation approaches produced different results in the 2D and 3D models. For the 2D model the *rounding after averaging* approach produced a substantially lower *Dice* metric than *rounding before averaging* approach. In the 3D model, the *rounding after averaging* approach produced an insignificantly higher *Dice* metric than *rounding before averaging*.

### 4.2. Non-medical Data (SpaceNet-Vegas)

**Figure 11:**
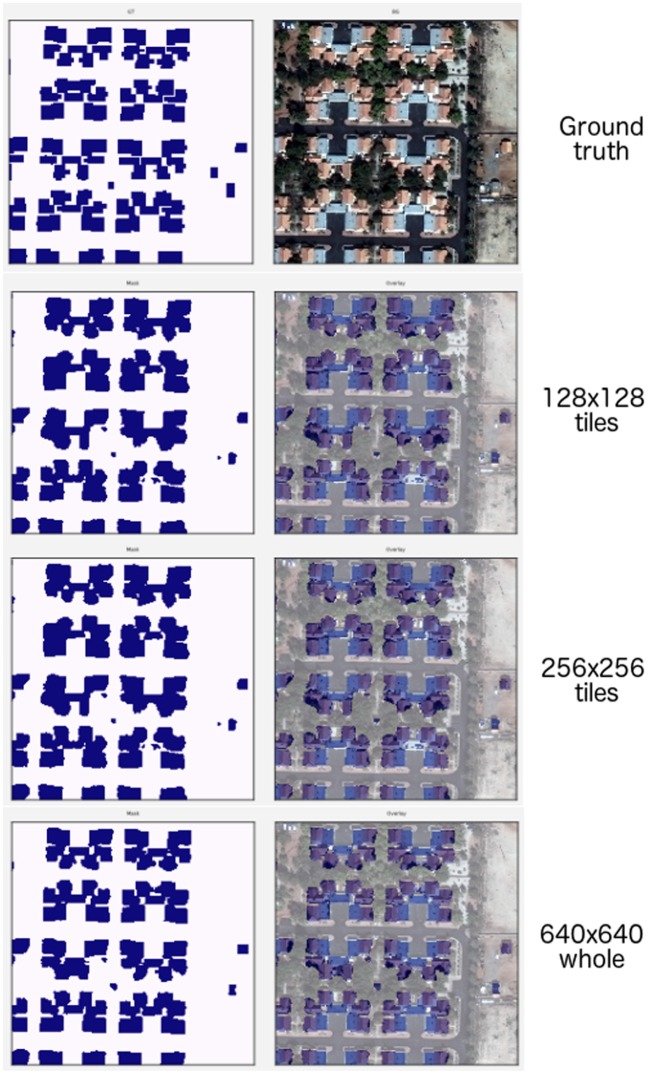
(1st row) Ground truth and whole image. (2nd row) 128 × 128 tiles. (3rd row) 256 × 256 tiles. (4th row) 640 × 640 whole image.

We find that the whole image consistently outperforms tiling-based approaches on the pixelwise *Dice* that converge to similar values once the tile size reached approximately half of the original height and width of the image (Table 3). Similarly, the polygonal-wise *Dice* (SpaceNet *F*1 metric) also improves as the training tiles cover a larger proportion of the whole image. Inspection of the predicted masks reveals the likely culprit: Figure 11 shows the ground truth mask and image at the top, followed by rows showing sliding window predictions with 128 × 128 and 256 × 256 tiles, with the last row being predictions from the model trained on 640 × 640 resized inputs.

We note that predictions from the models using smaller tile sizes produce segmentations that fail to capture fine-grained boundaries between buildings, leading to “blobbier” or more amorphous predictions. Note that the tiled predictions segment the buildings in each cul-de-sac as a single continuous mass; however, there are roughly 6 houses per cul-de-sac. These missing boundaries lead to the post-processing step of polygonization creating a reduced number of polygons as multiple buildings are getting extracted as one. We note that as the tile size increases to reach at least $\frac{1}{2}H\times \frac{1}{2}W$, then the adverse polygonization effects are reduced and predictions at the boundary of the segmentations becomes more accurate (Table 3).

The removal of zero padding in the topology has a negligible effect on the average per-image *Dice* coefficient. However, the *F*1 Metric is lower by 1–3% across all input size variants (Table 4). Because the removal of zero-padding reduces the output size of the model, what we see is an effect similar to the discussed effects of using smaller tiles rather than the whole image during training. Since the *F*1 metric is computed over the entirety of the extracted polygons—that is, a set latitude/longitude pairs defining a building footprint—we again lose fidelity at the edges of the buildings which decreased the SpaceNet *F*1 score. This does not affect the *Dice* score, as *Dice* is not sensitive to the separation of object instances. In other word, a giant pixel blob covering two buildings yields good pixelwise *Dice* values, but poor SpaceNet *F*1 polygon values.

## 5. Conclusions

In this study, we systematically evaluated the effects of using tiling approaches vs. using the whole image for deep learning semantic segmentation, in both 2D and 3D configurations. Through quantitative evaluation we demonstrated that larger tile (i.e., context) sizes yield more consistent results and mitigate undesirable and unpredictable behavior during inference. We realize that tiling methods may continue to be necessary as researchers use images with increasingly greater size and resolution in their convolutional neural network models. Our goal in this study is to raise awareness about the issues surrounding tiling. Namely:

1. Tiling hyperparameters, which include tile size, offset, orientation, and overlap, can cause large variations in the prediction, particularly around the borders of the segmentation mask.
2. This variance is not just limited to a translation less than the stride (as suggested by Huang et al., 2018), but seem to be present even with translations of ±2 in each direction. Therefore, we think that our results show a more complicated story to the translational variance of CNNs.
3. Topologies without zero padding in the convolutional layers do not eliminate the translational variance of the topology.
4. Methods to aggregate the individual predictions into a whole image prediction, namely when to average the predicted outcome pseudo-probability maps and when to round these predictions, that can have a significant effect on the overall accuracy.
5. Larger degrees of image context, including adding 3D information to the model and using larger tile sizes, improves model performance in training and is less sensitive to these hyperparameters during inference.

We conclude that increased access to memory—either through improvements in hardware or through high performance computing techniques, such as model parallelism (Shazeer et al., 2018) and data parallelism (Sergeev and Balso, 2018)—is essential to creating accurate and robust models. Tiling should only be reserved for those cases where the physical limitations of memory make it an absolute necessity. When tiling must be used, researchers should be careful to investigate how the translational variance of the model affects the predictions and compare methods of tiling aggregation to determine the best way to mitigate the variability inherent in tiling.

## Data Availability Statement

The datasets used for this study are publicly available as parts of the International Brain Tumor Segmentation challenge 2019 (https://www.med.upenn.edu/cbica/brats2019.html) and the SpaceNet satellite imagery dataset (https://spacenet.ai/spacenet-buildings-dataset-v2/). Appropriate citations for each of the datasets are provided within the article.

## Author Contributions

GR, RP, and SB: study conception and design. GR, RP, ST, and AB: software development used in the study. GR, RP, AB, and SB: wrote the paper. GR, RP, ST, AB, and SB: data analysis and interpretation, and reviewed/edited the paper.

### Conflict of Interest

GR, RP, and AB were employed by the company Intel Corporation. The remaining authors declare that the research was conducted in the absence of any commercial or financial relationships that could be construed as a potential conflict of interest.

## Footnotes

^1^www.med.upenn.edu/cbica/brats2019.html

^2^spacenet.ai/spacenet-buildings-dataset-v2/

^3^www.radiantsolutions.com

^4^github.com/IntelAI/unet

^5^inputs = tensorflow.keras.layers.Input([None, None, number_channels_in]).
